# Stimulation of P2Y11 receptor protects human cardiomyocytes against Hypoxia/Reoxygenation injury and involves PKCε signaling pathway

**DOI:** 10.1038/s41598-019-48006-6

**Published:** 2019-08-12

**Authors:** Lauriane Benoist, Stéphanie Chadet, Thibaud Genet, Claudie Lefort, Audrey Heraud, Maria D. Danila, Danina M. Muntean, Christophe Baron, Denis Angoulvant, Dominique Babuty, Thierry Bourguignon, Fabrice Ivanes

**Affiliations:** 1 EA4245 Transplantation, Immunologie et Inflammation, Loire Valley Cardiovascular Collaboration & Université de Tours, Tours, France; 2 Service de Chirurgie Cardiaque, Hôpital Trousseau, Centre Hospitalier Régional Universitaire de Tours, Tours, France; 3 Service de Cardiologie, Hôpital Trousseau, Centre Hospitalier Régional Universitaire de Tours, Tours, France; 4 Department of Pathophysiology - Functional Sciences, “Victor Babes” University of Medicine and Pharmacy, Timisoara, Romania; 5 Service de Néphrologie et d’Immunologie Clinique, Hôpital Bretonneau, Centre Hospitalier Régional Universitaire de Tours, Tours, France

**Keywords:** Cardiology, Medical research

## Abstract

Sterile inflammation is a key determinant of myocardial reperfusion injuries. It participates in infarct size determination in acute myocardial infarction and graft rejection following heart transplantation. We previously showed that P2Y11 exerted an immunosuppressive role in human dendritic cells, modulated cardiofibroblasts’ response to ischemia/reperfusion *in vitro* and delayed graft rejection in an allogeneic heterotopic heart transplantation model. We sought to investigate a possible role of P2Y11 in the cellular response of cardiomyocytes to ischemia/reperfusion. We subjected human AC16 cardiomyocytes to 5 h hypoxia/1 h reoxygenation (H/R). P2Y11R (P2Y11 receptor) selective agonist NF546 and/or antagonist NF340 were added at the onset of reoxygenation. Cellular damages were assessed by LDH release, MTT assay and intracellular ATP level; intracellular signaling pathways were explored. The role of P2Y11R in mitochondria-derived ROS production and mitochondrial respiration was investigated. *In vitro* H/R injuries were significantly reduced by P2Y11R stimulation at reoxygenation. This protection was suppressed with P2Y11R antagonism. P2Y11R stimulation following H_2_O_2_-induced oxidative stress reduced mitochondria-derived ROS production and damages through PKCε signaling pathway activation. Our results suggest a novel protective role of P2Y11 in cardiomyocytes against reperfusion injuries. Pharmacological post-conditioning targeting P2Y11R could therefore contribute to improve myocardial ischemia/reperfusion outcomes in acute myocardial infarction and cardiac transplantation.

## Introduction

Myocardial ischemia/reperfusion (I/R) is a pathologic process responsible for myocardial injuries observed in several common clinical situations. It is the central event in the pathophysiology of acute myocardial infarction, resulting in cardiomyocytes death, but also impacts the short- and long-term outcomes in heart transplantation, including chronic rejection.

Ischemia generates cellular stress leading to irreversible tissue injuries^1^. While shortening the ischemic period is mandatory to ensure cell survival and preserve organ function^2^, the bulky reintroduction of oxygen and nutrients associated with reperfusion generates excessive oxidative stress that further increases cellular damages and cardiomyocytes death^3^.

I/R injuries lead to the plasma membrane rupture and the release of endogenous danger molecules, also known as damage-associated molecular patterns (DAMPs), that bear the capacity to activate the immune system and promote the inflammatory response to I/R. DAMPs include extracellular ATP (eATP), an important immune regulator and a mediator of sterile inflammation^4^. Physiologically, eATP is locally released at low concentrations and plays the role of primary messenger in intercellular communications notably in the vascular system^5^. An important increase in eATP concentration is associated with cell damage and inflammatory processes^6^.

eATP activates purinergic receptors (P2Rs), divided into P2X ATP-gated ion channel receptors (P2X1-7) and G protein-coupled P2Y receptors (P2Y1, 2, 4, 6, 11–14). Both subtypes play important roles in immune cell functions, e.g. neutrophil migration^7^, inflammasome activation^8,9^ and dendritic cells (DCs) maturation^10^. DCs are sentinels that orchestrate both innate and adaptive immune response in the presence of DAMPs. They are also important immunoprotective regulators during post-myocardial infarction repair process by controlling monocytes/macrophages homeostasis^11^. We previously reported that eATP mediates human DCs maturation toward an immunosuppressive phenotype through P2Y11 receptor (P2Y11R) stimulation and inhibits Th1 polarization^12^.

P2Y11R is also present in cardiac and endothelial cells^13^. It was shown to be present in cardiofibroblasts, which don’t only play the role of support cells but also bear the capacity to modulate the local inflammatory response following I/R in a paracrine manner. Previous data from our group showed that cardiofibroblasts can exert a cardioprotective role in this context, notably when P2Y11R is stimulated^14^. In addition, we demonstrated that P2Y11R activation, in an *in vivo* murine model of heterotopic heart transplantation, could delay graft rejection through an attenuation of the local immuno-inflammatory response^15^, emphasizing a critical role of P2Y11 in the I/R-induced inflammatory response.

There are growing evidences that P2Y11R stimulation could have a protective role in myocardial I/R: Balogh *et al*. reported a positive inotropic effects of ATP in murine cardiomyocytes via P2Y11-like receptor signaling^16^; using a Langendorff rat heart model, Djerada *et al*. showed effective cardioprotection against I/R with extracellular NAAD pharmacological pre-conditioning involving P2Y11R-like^17^. Last, a P2Y11R polymorphism (Ala-87-Thr) was associated with an increase in both level of C-reactive protein and risk of myocardial infarction^18^. Thus, we hypothesized that pharmacological post-conditioning targeting the modulation of Gq/Gs protein-coupled P2Y11R may directly reduce I/R injuries. This could translate into significant improvements in post-myocardial infarction and post-transplantation outcomes, in addition to its already demonstrated effect on the post I/R inflammatory balance.

In this study, we report P2Y11R as a novel pharmacological post-conditioning target for cardioprotection against I/R through its modulation of oxidative stress in a human cardiomyocytes cell line.

## Results

### Hypoxia/reoxygenation induces AC16 cell death

**Figure 1:**
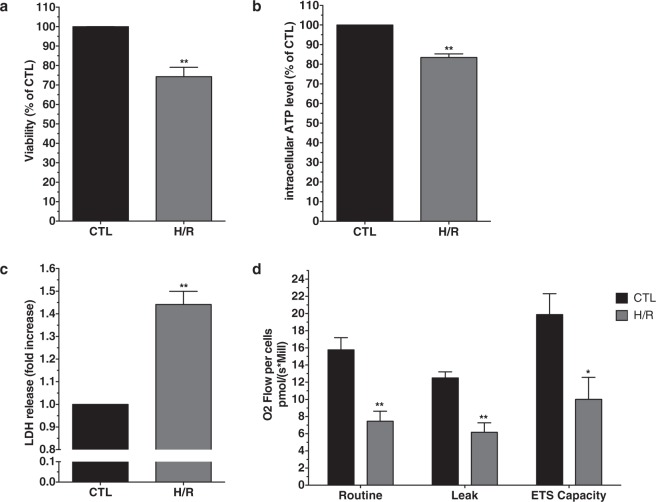
Hypoxia/Reoxygenation induced AC16 cells death. AC16 cells were subjected to hypoxia 5 h (1% O_2_, PBS) and reoxygenation 1 h (21% O_2_, DMEM) (H/R). (**a**) Viability (MTT) significantly decreased in H/R versus normoxic CTL, n = 8. (**b**) Intracellular ATP, a reflect of cell health, significantly decreased in H/R versus normoxic CTL, n = 8. (**c**) Cell death assessed by LDH release significantly increased in H/R versus normoxic CTL, n = 8. Data are given as relative mean ± s.e.m. (**d**) Oxygen consumption in non-permeabilized cells after 5 h/1 h H/R at basal state (Routine), state 4 (Leak) and maximal electron transport chain activity (ETS capacity) were significantly decreased compared to CTL. n = 4. ETS, electron transport system; *p < 0.05; **p < 0.01 from control condition (CTL).

We first examined whether 5 h/1 h of H/R could induce AC16 cardiomyocyte death. Cell viability assessed by MTT and intracellular ATP level was significantly reduced following H/R compared to normoxic CTL (−25.8% ± 4.8% and −16.6% ± 1.9% respectively, p < 0.01; n = 8) (Fig. 1a,b). This reduced viability was associated with a significant increase in LDH release compared to CTL (Fig. 1c) (1.44-fold ± 0.06, p < 0.01) (n = 8).

We then compared oxygen consumption in different energy states in non-permeabilized AC16 cells subjected to a 5 h/1 h H/R sequence and in normoxic CTL. Oxygen consumption in basal state was significantly decreased in H/R compared to normoxic CTL (7.45 ± 1.17 and 15.77 ± 1.43 pmol O_2_.min^−1^ per 10^6^ cells respectively, p < 0.05; n = 4) (Fig. 1d). In non-phosphorylating conditions (state 4, leak), oxygen consumption was significantly lower in H/R compared to normoxic CTL (6.17 ± 1.10 and 12.50 ± 0.70 pmol O_2_.min^−1^ per 10^6^ cells respectively, p < 0.05; n = 4). Maximal oxygen consumption measured in the presence of the uncoupling agent FCCP, reflecting maximal mitochondrial respiratory electron transport system capacity (ETS capacity) was also significantly lower in H/R compared to normoxic CTL (10.01 ± 2.56 and 19.87 ± 2.44 pmol O_2_.min^−1^ per 10^6^ cells respectively, p < 0.05; n = 4). These results demonstrated that AC16 cells exhibited decreased O_2_ consumption in different energy states following H/R.

### Extracellular ATP addition at reoxygenation provides cardioprotection to AC16 cells independently of adenosine receptors

**Figure 2:**
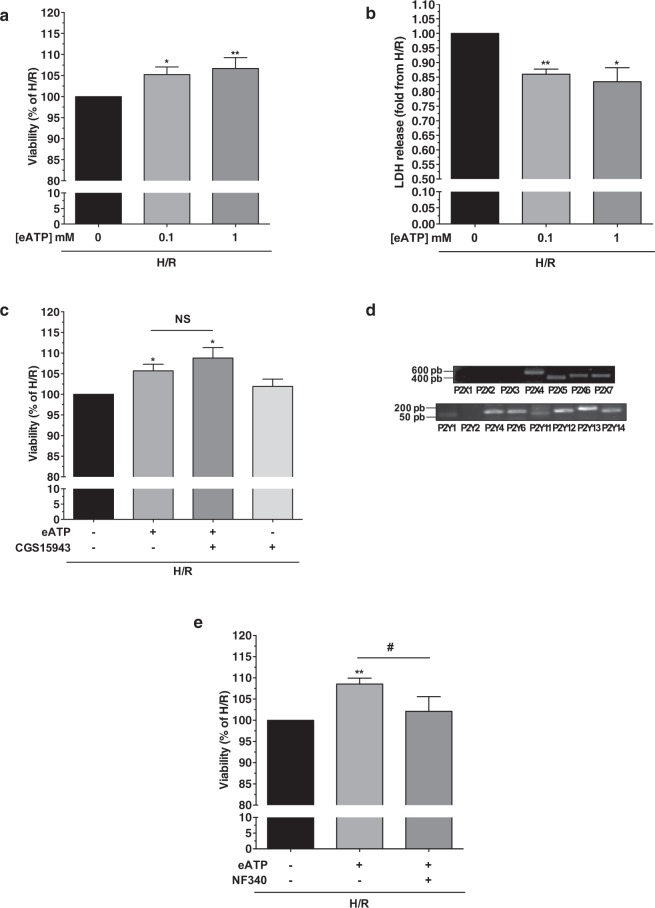
Addition of eATP at the onset of reoxygenation decreased AC16 cell death and involved P2Y11R. AC16 cells were subjected to 5 h/1 h H/R. P2Y11R modulators or vehicle were added at the time of reoxygenation. (**a**) Viability (MTT) significantly increased after ATP 0.1 and 1 mM compared to H/R condition, n = 8. (**b**) Necrosis assessed by LDH release significantly decreased after ATP 0.1 and 1 mM compared to H/R condition, n = 6. (**c**) Determination of the role of ATP hydrolysis into adenosine. CGS15943 3 µM (adenosine receptor antagonist) did not abolish the effect of ATP on cell viability (n = 7). Treatment with CGS15943 alone at the time of reoxygenation had no effect. n = 7. (**d**) mRNA expression for P2X and P2Y receptors (RT-PCR), n = 4. PCR images were cropped. For full-length images, see Supplementary Fig. 1. (**e**) Beneficial effect of ATP 1 mM on cell viability after H/R was significantly counteracted by P2Y11R antagonist NF340, n = 8. *p < 0.05; **p < 0.01; NS non significant from control condition (0/−); ^#^p < 0.05 comparison with eATP condition.

Addition of eATP 100 µM and 1 mM at the onset of reoxygenation significantly increased AC16 cells viability assessed by MTT (+5.3% ± 1.8% p < 0.05 and +6.7% ± 2.6% p < 0.01 respectively; n = 8) and decreased LDH release (−0.24-fold ±0.02 p < 0.01 and −0.27-fold ±0.05 p < 0.05 respectively; n = 6) (Fig. 2a,b). This effect was independent of ATP hydrolysis into adenosine as CGS-15943 (3 µM), an adenosine receptor antagonist, had no effect on cell viability (+8.8% ± 2.5% in presence of eATP and CGS-15943, p = NS; n = 7) (Fig. 2c).

### P2Y11 is responsible for eATP-mediated cardioprotection in AC16 cells

Considering the possible implication of P2Rs, we investigated their mRNA expression profile in AC16 cells using reverse transcription PCR. AC16 cardiomyocytes expressed mRNA for P2X4-7 and P2Y1, 4, 6, 11–14 (Fig. 2d). In light of the immunomodulatory role of P2Y11 in the eATP-mediated maturation of DC, we explored the effect of its selective antagonism on eATP-mediated cardioprotection. P2Y11R antagonist NF340 significantly abolished the beneficial effect of eATP on cell viability measured by MTT (+8.6% ± 1.4% *vs*. 2.1% ± 3.5%; p < 0.05; n = 8) (Fig. 2e). This suggests P2Y11R involvement in eATP-mediated cardioprotection following H/R.

### P2Y11R specific stimulation affords cardioprotection to AC16 cells against H/R injuries

**Figure 3:**
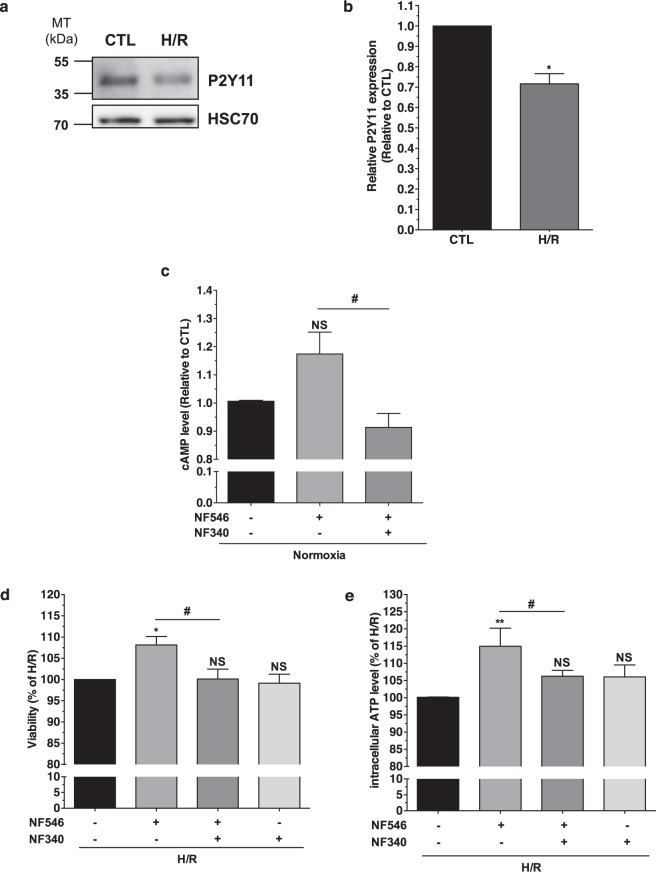
Specific stimulation of P2Y11R afforded cardioprotection to AC16 cells against Hypoxia/Reoxygenation injuries. (**a**) P2Y11R expression (western blot from total proteins) in AC16 cells. (**b**) P2Y11R expression (densitometric analysis) significantly decreased after H/R (n = 5). Blot images were cropped. For full-length images, see Supplementary Fig. 2. (**c**) Intracellular cAMP with P2Y11R agonist NF546 (10 µM) ± NF340, n = 3. (**d**) Viability (MTT) significantly increased with NF546 compared to H/R condition n = 5. (**e**) ATPi level significantly increased with NF546, NF340 abolished this effect, n = 5. *p < 0.05; **p < 0.01; NS non significant from control condition (CTL/−); ^#^p < 0.05 versus NF546 condition.

P2Y11R is present in AC16 cells, although H/R significantly decreased its expression (−28% ± 5%; p < 0.05, n = 5) (Fig. 3a,b). P2Y11R being the only P2YR able to activate Gs protein signaling, we tested the specificity of NF546 and NF340 regarding cAMP production. NF546 increased cAMP level (from 1 to 1.17 ± 0.08; p = 0.125 vs CTL) and NF340 abolished this effect (0.91 ± 0.05; p < 0.05 *vs*. NF546 condition; n = 3) (Fig. 3c), suggesting that P2Y11R is fully functional in AC16 cells. When added at the onset of reoxygenation, NF546 (10 µM) significantly increased H/R AC16 cell viability (+8.1% ± 2.0%, p < 0.05, and +15.0% ± 5.3%, p < 0.01, for MTT and intracellular ATP level assays respectively, n = 5 each) (Fig. 3d,e). This protection was suppressed by NF340 (10 µM) demonstrating a specific cardioprotective role of P2Y11. Addition of NF340 alone did not worsen cell viability, suggesting that ATP released by stressed AC16 cells did not act in an autocrine/paracrine manner on P2Y11R.

### P2Y11R stimulation protects AC16 cells against oxidative stress-induced lesions

**Figure 4:**
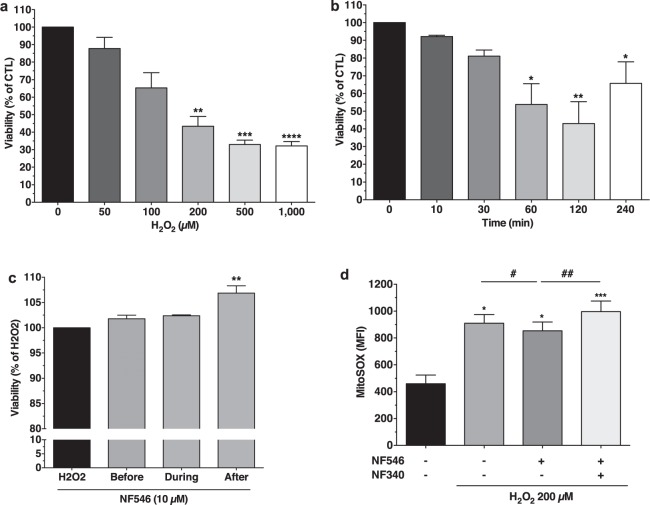
P2Y11R stimulation protected AC16 cells against H_2_O_2_-induced oxidative stress and decreased mitochondrial ROS production. (**a**) Viability (MTT) of AC16 cells after H_2_O_2_ (2 h, increasing concentrations) was significantly reduced in a dose-dependent manner n = 7. (**b**) Viability (MTT) after H_2_O_2_ (200 µM, different times), n = 4. (**c**) Effect of NF546 treatment (1 h) before, during or after H_2_O_2_ (2 h, 200 µM). Post-stimulation of P2Y11R significantly increased viability assessed by MTT, n = 6. (**d**) Mitochondrial ROS production (MitoSOX staining, flow cytometry analysis) after H_2_0_2_ 30 min and a 1 h-treatment with NF546 and/or NF340. Results are expressed as mean fluorescence intensity (MFI). Mitochondrial ROS production significantly decreased with P2Y11R stimulation and increased when P2Y11R was antagonized, n = 6. *p < 0.05; **p < 0.01; ***p < 0.001; ****p < 0.0001; NS non significant from control condition (0/−/H_2_O_2_); ^#^p < 0.05; ^##^p < 0.01 versus H_2_O_2_ + NF546 condition.

Oxidative stress plays an important role in H/R injuries. Exposure of AC16 cells to H_2_O_2_ (50 µM–1 mM) during 2 h reduced cell viability in a dose-dependent manner (Fig. 4a). H_2_O_2_ 50 µM and 100 µM had little effect while 200 µM, 500 µM and 1 mM significantly decreased cell viability (−43.4% ± 5.6%, p < 0.01; −33.0% ± 2.5%, p < 0.001; −32.2% ± 2.5%, p < 0.0001 respectively compared to CTL, n = 7) without additional effects of longer exposure to H_2_O_2_ (Fig. 4b). Consequently, we used H_2_O_2_ 200 µM for subsequent experiments.

AC16 cells were treated with NF546 for 1 h before, during, or after a 2 h H_2_O_2_ exposure. Only P2Y11R post-stimulation significantly increased cell viability (+7% ± 1.4%, p < 0.01; n = 6) (Fig. 4c).

### P2Y11R stimulation reduces H2O2-induced mitochondrial ROS production and activates PKCε signaling pathway, but has no impact on mitochondrial respiration

AC16 cells were subjected to H_2_O_2_ (30 min) and mitochondrial ROS production was evaluated by flow cytometry using MitoSOX™ dye. Oxidative stress significantly increased MitoSOX™ fluorescence (MFI from 459.8 ± 64.3 to 909.8 ± 65.5, n = 6) (Fig. 4d). NF546 treatment during 1 h following H_2_O_2_ exposure significantly decreased the signal (MFI 853.5 ± 64.8; p = 0.0313 *vs*. H_2_O_2_, n = 6). This effect was abolished in case of co-treatment with NF340 (MFI 996.7 ± 78.5; p < 0.01 *vs*. H_2_O_2_ + NF546) suggesting an anti-oxidative role of P2Y11R.

**Figure 5:**
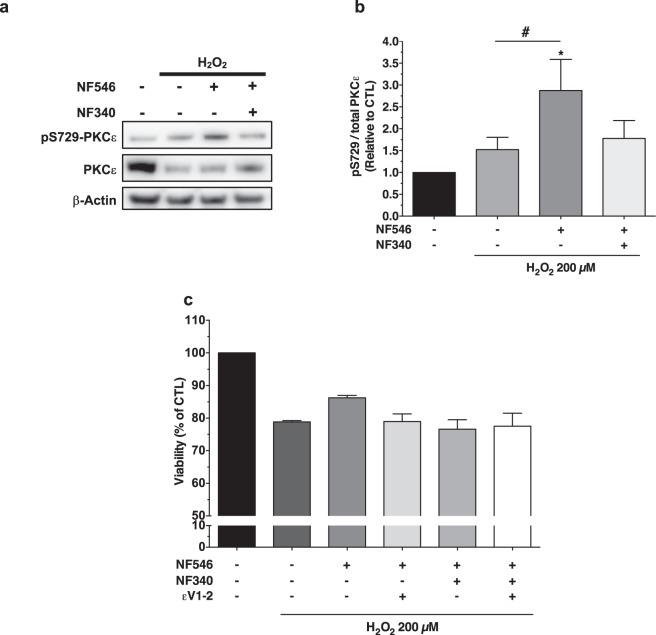
P2Y11R-mediated cardioprotection against H_2_O_2_-induced oxidative stress involved PKCε signaling pathway. (**a**,**b**) PKCε phosphorylation in S729 (western blot) after H_2_O_2_ 30 min. Blot images were cropped. For full-length images, see Supplementary Fig. 3. Results expressed as pS729/total PKCε relative to β-Actin versus CTL condition. PKCε activation was significantly higher when P2Y11R was post-stimulated, n = 6. (**c**) Treatment with NF546 (1 h) after H_2_O_2_ (2 h) had no more effect on cell viability (MTT) when AC16 cells were pre-treated with PKCε translocation inhibitor Peptide εV1-V2 (10 µM). There was no additional effect of NF340 treatment in combination with NF546 + εV1-V2 suggesting a pivotal role of PKCε in P2Y11R-mediated cardioprotection, n = 3. *p < 0.05 from control condition (−); ^#^p < 0.05 versus H_2_O_2_ condition.

Reperfusion Injury Salvage Kinases (RISK) and Survivor Activating Factor Enhancement (SAFE) signaling pathways are both pro-survival kinases cascades targeted in cardioprotective response strategies that share downstream activation of protein kinase C ε isoform (PKCε). A 30 min treatment with NF546 following oxidative stress significantly increased the Ser729-phosphorylated form of PKCε in western blot (2.8-fold ± 0.7 *vs*. CTL, p < 0.05; n = 6) (Fig. 5a,b). Again, co-treatment with NF340 abolished this effect, suggesting a downstream regulation of PKCε signaling pathway by P2Y11R stimulation that may play a key role in this cardioprotective effect.

To go further into the mechanistic assessment of the cardioprotective role of P2Y11R through PKCε signaling pathway activation, we evaluated the effect of the selective PKCε translocation peptide inhibitor εV1-2 on AC16 cells viability subjected to H_2_O_2_ in the presence of P2Y11R modulators (Fig. 5c). While treatment with NF546 following oxidative stress increased cell viability (78.9% ± 0.4% *vs*. 86.3% ± 0.7%; H_2_O_2_ and H_2_O_2_ + NF546 respectively, compared to CTL), co-treatment with εV1-2 appeared to abolish this effect (78.9% ± 2.3%; n = 3). NF340 antagonized NF546 protective effect on oxidative stress (76.6% ± 2.9%), but triple treatment with NF546, NF340 and εV1-2 did not appear to further decrease AC16 cells viability (77.5% ± 3.9%; n = 3), suggesting a pivotal role of PKCε in P2Y11R-mediated cardioprotection.

We finally investigated the impact of P2Y11R on mitochondrial oxygen consumption. Modulation of P2Y11R with its specific agonist/antagonist had no effect on mitochondrial respiration following H/R, whatever the state (data not shown), suggesting that the protective effect of P2Y11R activation cannot be explained by a specific effect on the mitochondrial respiratory chain and oxygen consumption mechanisms.

## Discussion

In the present study, we demonstrated a novel cardioprotective role of P2Y11. Pharmacological post-conditioning with selective stimulation of P2Y11R rescued AC16 cardiomyocytes viability after H/R.

We propose AC16 human cardiomyocytes as a new relevant *in vitro* model to study H/R injuries. Oxygen-nutrients deprivation and resupply decreased AC16 cells viability, increased LDH-assessed necrosis and impaired mitochondrial respiration. Suffering cardiac cells during I/R and particularly cardiomyocytes activate multiple cell death pathways leading to the release of danger signals or DAMPs, among which extracellular ATP^19–22^ has been identified as an important modulator of immune system through P2Rs signaling^4,6,23^. We and others previously reported that eATP through a P2Y11R-dependent signaling induced DCs maturation towards a tolerogeneic phenotype leading to modulation of effector T cells activation by the inhibition of Th1 response^10,12,24,25^. We demonstrated here a cardioprotective post-conditioning-like effect of eATP against H/R injuries. The cardioprotective effects of the hydrolysed form of ATP is now well admitted^26^. Adenosine receptors were associated to the cardioprotective effect of ischemic preconditioning^27^ and they showed I/R injuries reduction properties when activated at reperfusion^28^. The use of an adenosine receptor antagonist here strongly suggests that the beneficial effect of eATP mainly involved P2R signaling.

P2Rs are expressed in different cell types of the cardiovascular system and their involvement in cardiac physiology recently emerged. Besides the positive inotropic effects of ATP, UTP and UDP on cardiomyocytes through P2Y11R, P2Y2R and P2Y6R activation respectively^13,16,29^, there are growing evidence that P2Rs play key roles in the physiopathology of I/R. Wee *et al*. reported a significant worsening in ischemic tolerance and post-ischemic outcomes due to P2Rs antagonism with suramin in a murine Langendorff model^30^. Moreover, both ATP and UTP released during cardiac ischemia displayed cardioprotective effects in several rodent model studies^30,31^. But because of the lack of selective agonists/antagonists until recently and the differences in pharmacological properties between human and rodent P2Rs, the receptors involved in such effects were not clearly identified.

NF546 has been identified by Meis *et al*.^32^ as a relatively selective, non-nucleotide agonist for P2Y11R over other P2YR, with a competitive behaviour toward the nanomolar potency antagonist NF340. Using NF546, we are the first to show that selective P2Y11R stimulation at the onset of reoxygenation protected human AC16 cardiomyocytes from H/R injuries. This is in line with Djerada *et al*. who reported that rat hearts treated with NAADP before I/R sequence were protected against I/R injuries through P2Y11R activation in a Langendorff model^17^. In the publication of Amisted *et al*. reporting an increased risk of myocardial infarction associated with a P2Y11R polymorphism in residue 87, the suggested mechanisms were probable impairments in ligand binding and signaling^18^.

Regarding the molecular mechanisms involved in the cardioprotective effect of P2Y11R, we showed that its stimulation following H_2_O_2_-induced oxidative stress increased cell recovery through reduction of mitochondrial ROS production. Our data suggest that this effect was related to an increased PKCε phosphorylation. This is particularly relevant as PKCε can be activated by phospholipase C/diacylglycerol signaling pathway following Gq protein-coupled receptors stimulation (e.g. by P2Y11R), enabling its importation to mitochondria where it exerts its cardioprotective effect. In line with this, our data suggest that the inhibition of PKCε translocation to mitochondria abolished P2Y11R-mediated cardioprotection following H_2_O_2_-induced oxidative stress, with no additional effect of P2Y11R antagonist. These results support a close association between P2Y11R stimulation and PKCε signaling pathway in transducing cardioprotective signal. In our experiments, we couldn’t link PKCε activation to a modulation of mitochondrial oxygen metabolism. Yet, modulation of oxidative phosphorylation is not the sole-described effect of PKCε signaling pathway. PKCε is considered to play a critical part in cardioprotection due to its ability to interact with substantial mitochondrial proteins, e.g. extracellular signal-regulated kinases (ERK) or ATP-sensitive K+ channels thereafter modifying mitochondrial permeability transition^33^.

All our data strongly suggest that P2Y11 may modulate myocardial H/R injuries. Limiting both I/R injuries and oxidative stress through P2Y11R stimulation might indirectly influence the inflammatory response (by decreasing the release of DAMPs and ROS), which could be synergistic with the effects of P2Y11R stimulation on DCs as previously described *in vitro*^12^ and *in vivo*^15^. Of note, Certal *et al*. reported an anti-proliferative effect of P2Y11 in cardiac myofibroblasts, suggesting that this receptor may also be an interesting target to modulate cardiac remodeling^34^. We recently reported a similar anti-fibrotic effect of P2Y11R activation in cardiac fibroblasts that also displayed an immunomodulatory and cardioprotective role^14^. A limitation of this study has to do with the characteristic of AC16 cells, which is a proliferating cardiomyocyte cell line. Though they have retained the nuclear and mitochondrial DNA of the primary cardiomyocytes, cellular metabolism and proliferative status are correlates and both could modify response to I/R. As a reminder, we obtained similar data using the well-characterized H9c2 cardiomyocytes cell line^15^. Yet, with regards to this point, the effect of P2Y11 on primary cardiomyocytes remains speculative and needs to be confirmed. Nevertheless, the protective effect of P2Y11R previously reported regarding inhibition of graft rejection on transplanted hearts could be promising as to his role on non-proliferative cardiomyocytes.

The next step to demonstrate the therapeutic relevance of this approach, i.e. modulation of P2Y11R, in the context of myocardial ischemia/reperfusion should be through an *in vivo* model of acute myocardial infarction, with the additional advantage to validate the concept on non-proliferative cardiomyocytes.

In conclusion, our results propose a novel protective role of P2Y11R as a pharmacological post-conditioning target through a reduction of myocardial I/R injuries. This property combined with our previous observations of immunomodulatory and anti-fibrotic effects support the idea that therapeutic interventions aiming at stimulating P2Y11R could provide beneficial effects in the setting of acute myocardial infarction and cardiac transplantation and improve patients’ outcomes.

## Methods

### Reagents

ATP, CGS-15943, H_2_O_2_, Thiazolyl Blue Tetrazolium Bromide (MTT) and chemicals were purchased from Sigma-Aldrich, P2Y11R agonist NF546 and antagonist NF340 from R&D systems, PKCε translocation inhibitor peptide εV1-2 (EAVSLKPT) from Santa Cruz Biotechnologies. Drugs were prepared in Gibco® Dulbecco’s Phosphate Buffered Saline (PBS) with CaCl_2_ and MgCl_2_ from Fisher Scientific.

### Cell culture

AC16 cells were purchased from American Type Culture Collection (ATCC®, LGC Standards) and cultured in Gibco® Dulbecco’s modified Eagle’s medium: Nutrient mixture F-12 (DMEM/F12) supplemented with 10% foetal bovine serum (FBS) (HyClone™, GE Healthcare Life Sciences) and Penicillin-Streptomycin (100 U/mL, Gibco®) in a humidified incubator at 37 °C with 5% CO_2_.

### *In vitro* hypoxia/reoxygenation (H/R)

H/R was simulated *in vitro* by oxygen and nutrient deprivation as previously described^12,14,15^. H/R cells were placed in PBS with CaCl_2_ and MgCl_2_ in a hypoxic chamber (INVIVO_2_ 200, Ruskinn Technology) at 37 °C with 1% O_2_, 94% N_2_ and 5% CO_2_ for 5 h and then reoxygenated in a normoxic humidified incubator at 37 °C for 1 h in FBS-free Gibco® Dulbecco’s modified Eagle’s medium (DMEM, GE Healthcare Life Sciences) with vehicle or treatments. These hypoxia/reoxygenation durations were considered following previous exploratory experiments where different hypoxia and reoxygenation times were tested as to obtain the best experimental conditions to explore the cardioprotective effect of P2Y11R stimulation (data not shown). Control cells (CTL) were left in FBS-free DMEM in a normoxic humidified incubator at 37 °C for the same durations.

### H_2_O_2_-induced oxidative stress

AC16 cells were FBS-deprived 1 h before the oxidative stress sequence. Oxidative stress was induced by H_2_O_2_ (50 µM–1 mM in DMEM) for the indicated time. Then, cells were re-placed in fresh DMEM for 1 h. Treatments were added before, during or after the oxidative stress sequence for the indicated duration.

For PKCε inhibition experiments, AC16 cells were transiently permeabilized with digitonine (7,5 µg/mL) with or without the selective PKCε translocation inhibitor εV1-2 (10 µM) for 1 min prior to oxidative stress sequence.

### Cardiomyocytes viability and death assessments

MTT reduction and CellTiter-Glo® Luminescent Assay (Promega) measuring intracellular ATP production were used as indicators of cell viability.

Following H/R or oxidative stress sequences, cells were incubated for 1 h with a MTT solution (0.5 mg/mL in DMEM with 5% FBS) at 37 °C in a humidified incubator with 5% CO_2_. The resulting formazan produced by viable mitochondria was solubilized in DMSO for 45 min. Absorbance was recorded at 550 nm using a microplate reader.

CellTiter-Glo® experiments were performed according to manufacturer’s instructions using CellTiter-Glo® diluted in PBS with CaCl_2_ and MgCl_2_ (1:1) added directly to cells in culture for 10 min at 37 °C. Resulting luminescence was measured with a GloMax®-Multi Jr luminometer (Promega).

LDH activity, a surrogate marker of necrosis, was measured in cardiomyocytes supernatant using the *In Vitro* Toxicology Assay Kit (Sigma) according to manufacturer’s instructions. At 30 min, final absorbance was measured at 490 nm.

### High resolution respirometry

Oxygen consumption was measured in non-attached AC16 cells resuspended in DMEM without FBS (2.5 × 10^6^ cells/ml) using a 2 mL chamber OROBOROS® Oxygraph 2 K (Oroboros Instruments, Innsbruck, Austria) at 37 °C. Respiration rates were calculated as the time derivative of oxygen concentration measured in the closed respirometer, expressed per million viable cells and corrected by non-mitochondrial oxygen consumption (energy wasting process) measured with antimycin A (2 μM). Oxygen consumption was measured in intact cells following 5 h of hypoxia and 1 h of reoxygenation at basal state, state 4 (non-phosphorylating state) using oligomycin B (10 μg/mL) and ETS (Electron Transfer System) capacity (maximum uncoupled respiration) induced by Carbonyl cyanide-4-(trifluoromethoxy)phenylhydrazone (FCCP, 0.8 µM). Results were expressed as O_2_ flow (pmol.min^−1^) per million cells.

### Mitochondria-derived ROS detection

Mitochondrial superoxide anion production was measured using MitoSOX™ Red probe (Invitrogen). Following oxidative stress sequence, AC16 cells were incubated with 5 µM MitoSOX™ Red in FBS-free DMEM for 10 min. Mean fluorescence intensity (MFI) was then analysed with a BD™ FACSCanto™ I flow cytometer (BD™ Biosciences).

### Non-quantitative polymerase chain reaction

Total RNA was extracted from cardiomyocytes using the Illustra RNAspin mini kit (GE Healthcare Life Sciences) and reverse transcribed using the Protoscript® II Reverse Transcriptase (New England Biolabs) according to manufacturer’s instruction. cDNA were subjected to 40 cycles of amplification using OneTaq® DNA Polymerase (New England Biolabs) and specific primers targeting human P2X1-7 and P2Y1, 2, 4, 6, 11–14 receptors (see Table 1) as previously described^12^ with modified Tm. PCR products were separated on 2% Agarose gels containing 1/10,000 SYBR® Safe DNA Gel Stain (Invitrogen) and revealed under UV light with a PXi/PXi Touch gel imaging system and UltraSlim blue LED transilluminator (Syngene).

**Table 1:** Human P2R primers for non-quantitative PCR.

Gene	Forward primer	Reverse primer	Amplification size (pb)	Tm
P2X1R	TTTCATCGTGACCCCGAAGCAG	TCAAAGCGAATCCCAAACACC	633	58 °C
P2X2R	ACCTGCCCCGAGAGCATAAG	AATGACCCCGATGACACCACCC	426	58 °C
P2X3R	CACCTCGGTCTTTGTCATCATCAC	TGTTGAACTTGCCAGCATTCC	695	58 °C
P2X4R	ACAGCAACGGAGTCTCAACAGG	CCTTCCCAAACACAATGATGTCG	561	58 °C
P2X5R	AACCTGATTGTGACCCCCAACC	TCGCAGAAGAAAGCACCCTTGC	683	58 °C
P2X6R	GGTGACCAACTTCCTTGTGACG	CCCAGTGAACTCTGATGCCTACAG	476	58 °C
P2X7R	TGCGATGGACTTCACAGATTTG	TGCCCTTCACTCTTCGGAAAC	465	58 °C
P2Y1R	CCGGCTGTCTACATCTTGGT	GGCAGAGTCAGCACGTACAA	152	62 °C
P2Y2R	CCACCTGCCTTCTCACTAGC	TGGGAAATCTCAAGGACTGG	163	62 °C
P2Y4R	TGCCTGGTCACTCTTGTTTG	GTACTCGGCAGTCAGCTTCC	205	62 °C
P2Y6R	CGACCACATGAGCTCCTACA	GAGCTTCTGGGTCCTGTGAG	198	62 °C
P2Y11R	TAGCAGACACAGGCTGAGGA	CACCAGGAACTCAACCACCA	156	62 °C
P2Y12R	AACTGGGAACAGGACCACTG	ACATGAATGCCCAGATGACA	201	62 °C
P2Y13R	TCGTGGCTGTCTTCTTTGTG	TTTCTTGGCTTGATGCTGTG	249	62 °C
P2Y14R	TTAAAAGGCCTCTGCCTTCA	AGAGCTGGGCACGTAAAAGA	190	62 °C

### Western blot

AC16 cells were lysed with RIPA lysis buffer containing 1% Triton X-100 and proteases inhibitors cocktail (Sigma-Aldrich) for 1 h at 4 °C. Samples (50 µg proteins) were loaded to Bolt™ 4–12% Bis-Tris Plus gels (Invitrogen) and transferred to Amersham™ Hybond™ PVDF membranes (GE Healthcare Life Sciences). The primary antibodies (see Table 2) were incubated overnight at 4 °C. Membranes were further incubated for 1 h at room temperature with appropriate secondary antibodies. Proteins were detected with Amersham™ ECL™ Prime reagent (GE Healthcare Life Sciences) using a PXi/PXi Touch gel imaging system and bands were quantified by densitometric analysis using Fiji software (distributed by ImageJ)^35^. Of note, for each experiment, proteins of every conditions were loaded in the same gel and transferred to a membrane that was then cut in order to incubate appropriate antibodies. Densitometric analyses of bands were calculated as relative to control protein loading (e.g. HSC70 or ß-Actin).

**Table 2:** Antibodies for Western Blot.

Primary antibodies	Supplier	Species	Type	Reference
P2Y11R	Alomone	Rabbit	Polyclonal	#APR-015
PKCε	Santa Cruz Biotechnology	Rabbit	Polyclonal	Sc-214
Phospho(Ser729)-PKCε	Abcam	Rabbit	Polyclonal	Ab63387
HSC70	Santa Cruz Biotechnology	Mouse	Monoclonal	Sc-7298
β-Actin-HRP conjugate	Cell Signaling Technology	Mouse	Monoclonal	#12262
**Secondary antibodies**	**Supplier**	**Species**	**Type**	**Reference**
Rabbit-HRP conjugate	Biorad	Goat	Polyclonal	#170-6515
Mouse-HRP conjugate	Biorad	Goat	Polyclonal	#170-6516

### Intracellular cAMP quantification

Following a 15 min treatment with NF546 10 µM and/or NF340 10 µM in FBS-free DMEM, cAMP production in AC16 cells was quantified using the luminescent cAMP-Glo™ Assay kit (Promega) according to manufacturer’s instructions. Luminescence values were normalized to control (without agonist/antagonist). Data were expressed as 1/mean relative to vehicle.

### Statistical analyses

Results are expressed as mean ± s.e.m. Comparisons were performed by Wilcoxon test, Friedman test followed by Dunnett’s multiple comparisons test using GraphPad Prism 5.0 f for Mac OS X. A *p*-value < 0.05 was considered significant.

## Supplementary information

Supplementary information

## Data Availability

All data regarding this work is made available by the authors.
