# Supplementary material for: Stimulation of P2Y11 receptor protects human cardiomyocytes against Hypoxia/Reoxygenation injury and involves PKCε signaling pathway
Source: Sci Rep. 2019 Aug 12;9:11613. doi: 10.1038/s41598-019-48006-6 (PMC6690895; doi:10.1038/s41598-019-48006-6)
Supplement: Supplementary file 1 — Supplementary information [file 41598_2019_48006_MOESM1_ESM.pdf]

# Stimulation of P2Y11 receptor protects human cardiomyocytes against Hypoxia/Reoxygenation injury and involves PKCε signaling pathway

Lauriane Benoist[1], Stéphanie Chadet[1], Thibaud Genet[3], Claudie Lefort[1], Audrey Heraud[1], Maria D. Danila[4], Danina M. Muntean[4], Christophe Baron[1,5], Denis Angoulvant[1,3], Dominique Babuty[3], Thierry Bourguignon[1,2], Fabrice Ivanes[1,3*]

[1]EA4245 Transplantation, Immunologie et Inflammation, Loire Valley Cardiovascular Collaboration & Université de Tours, Tours, France; [2]Service de Chirurgie Cardiaque, Hôpital Trousseau, Centre Hospitalier Régional Universitaire de Tours, Tours, France; [3]Service de Cardiologie, Hôpital Trousseau, Centre Hospitalier Régional Universitaire de Tours, Tours, France; [4]Department of Pathophysiology - Functional Sciences, "*Victor Babes*" University of Medicine and Pharmacy, Timisoara, Romania; [5]Service de Néphrologie et d'Immunologie Clinique, Hôpital Bretonneau, Centre Hospitalier Régional Universitaire de Tours, Tours, France

**Supplementary figure 1**

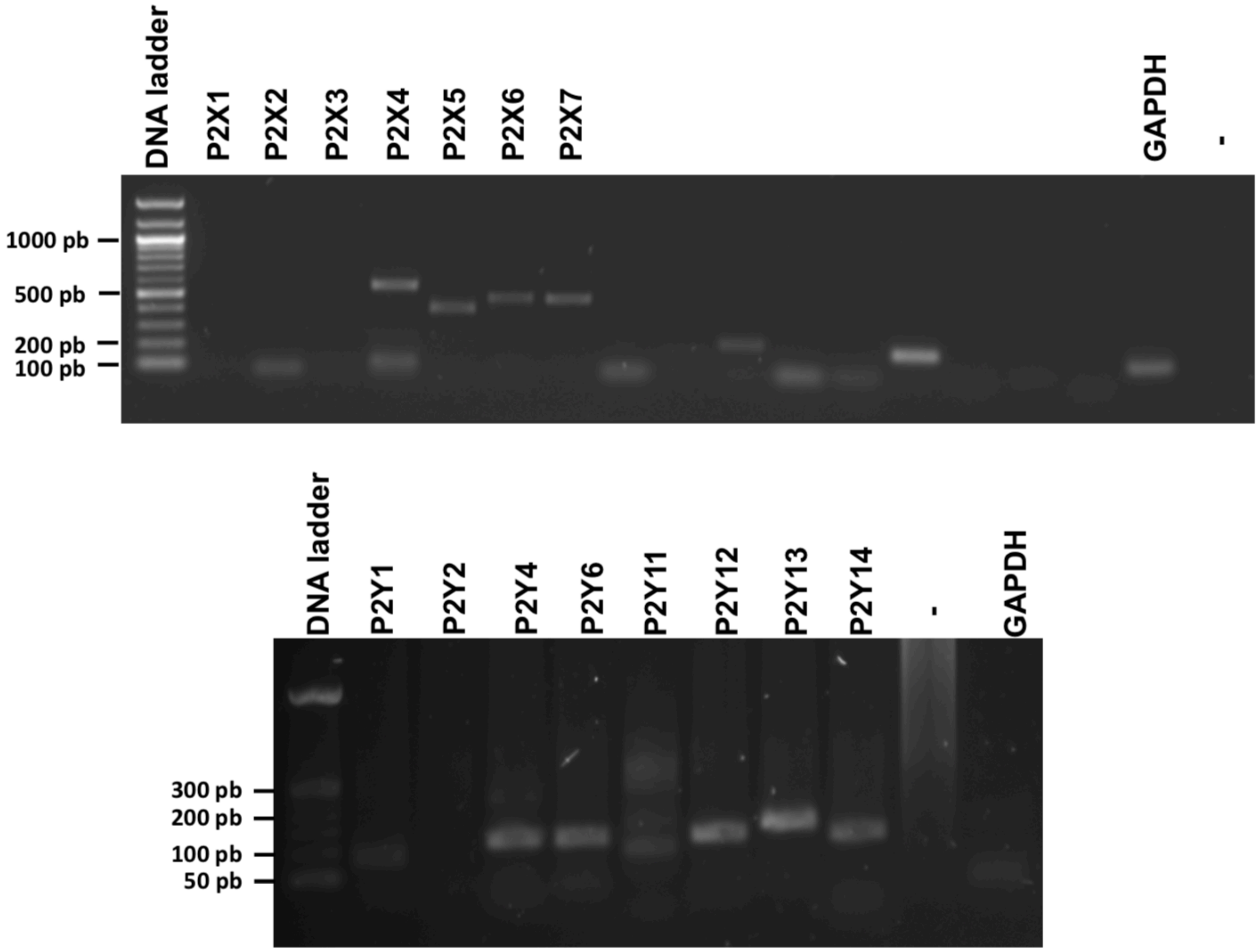


Supplementary figure 1. mRNA expression for P2X and P2Y receptors (RT-PCR), n=4

**Supplementary figure 2**

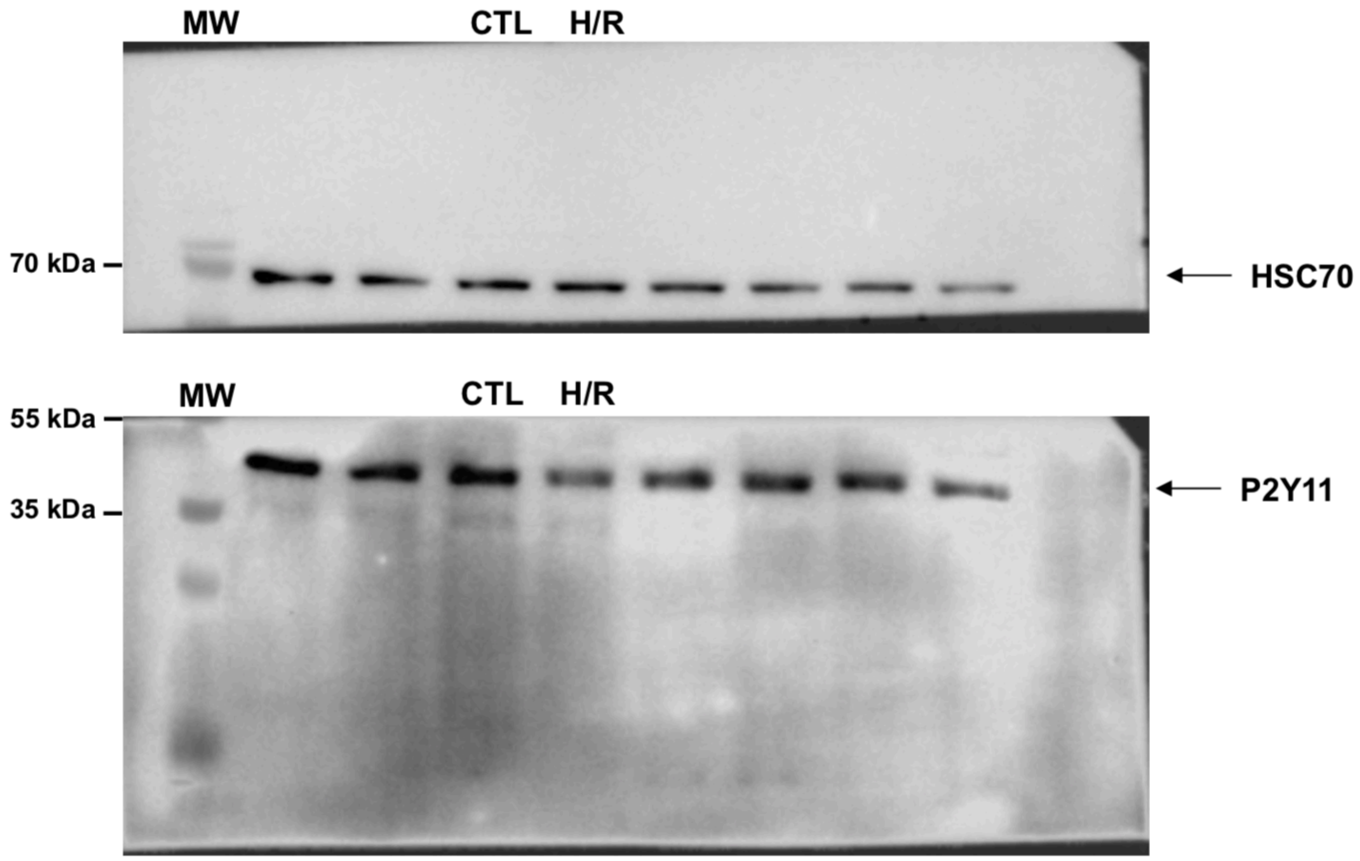


Supplementary figure 2. P2Y11R expression (densitometric analysis) significantly decreased after H/R (n=5).

## Supplementary figure 3

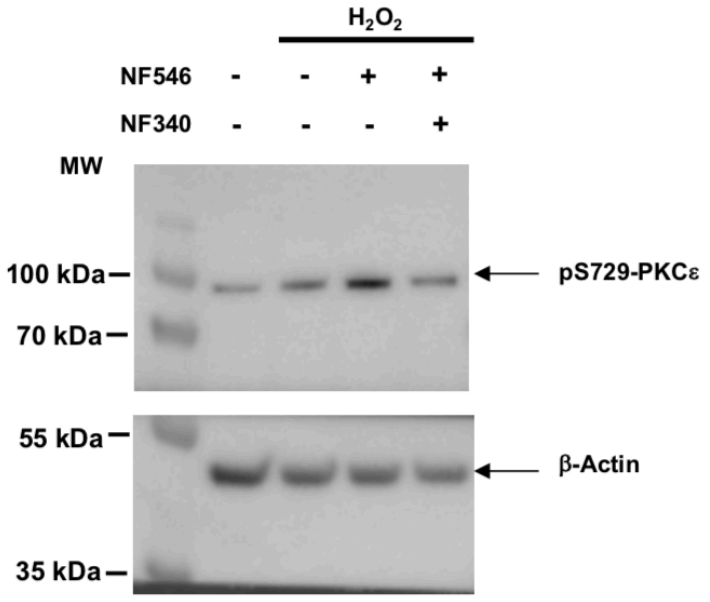


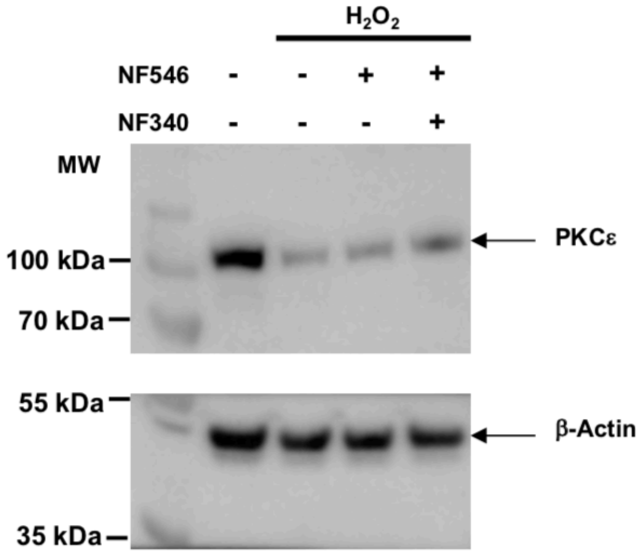


Supplementary figure 3. PKCε phosphorylation in S729 (western blot) after $H_2O_2$ 30 min. (n=6)
